# Novel bispecific nanobody mitigates experimental intestinal inflammation in mice by targeting TNF‐α and IL‐23p19 bioactivities

**DOI:** 10.1002/ctm2.1636

**Published:** 2024-03-27

**Authors:** Jiewen Wang, Guangbo Kang, Huiying Lu, Ario de Marco, Haibin Yuan, Zelin Feng, Mengxue Gao, Xiaoli Wang, Huahong Wang, Xiaolan Zhang, Yuli Wang, Miao Zhang, Ping Wang, Yuanhang Feng, Zhanju Liu, Xiaocang Cao, He Huang

**Affiliations:** ^1^ Frontiers Science Center for Synthetic Biology and Key Laboratory of Systems Bioengineering (Ministry of Education), School of Chemical Engineering and Technology Tianjin University Tianjin China; ^2^ Center for Inflammatory Bowel Disease Research and Department of Gastroenterology, The Shanghai Tenth People's Hospital Tongji University School of Medicine Shanghai China; ^3^ Laboratory for Environmental and Life Sciences University of Nova Gorica Nova Gorica Slovenia; ^4^ Department of Gastroenterology and Hepatology, Tianjin Medical University General Hospital Tianjin Medical University Tianjin China; ^5^ Department of Gastroenterology Peking University First Hospital Beijing China; ^6^ Department of Gastroenterology The Second Hospital of Hebei Medical University Shijiazhuang China; ^7^ Tianjin Pharmaceutical Da Ren Tang Group Corporation Limited, Traditional Chinese Pharmacy Research Institute Tianjin Key Laboratory of Quality Control in Chinese Medicine Tianjin China; ^8^ State Key Laboratory of Drug Delivery Technology and Pharmacokinetics Tianjin Institute of Pharmaceutical Research Tianjin China; ^9^ China Resources Biopharmaceutical Company Limited Beijing China; ^10^ New Technology R&D Department Tianjin Modern Innovative TCM Technology Company Limited Tianjin China

**Keywords:** anti‐TNF‐α mAb, bispecific nanobodies, inflammatory bowel disease, TNFR2^+^IL23R^+^ T cells, VHH‐Fc

## Abstract

**Background:**

Inflammatory bowel diseases (IBDs) pose significant challenges in terms of treatment non‐response, necessitating the development of novel therapeutic approaches. Although biological medicines that target TNF‐α (tumour necrosis factor‐α) have shown clinical success in some IBD patients, a substantial proportion still fails to respond.

**Methods:**

We designed bispecific nanobodies (BsNbs) with the ability to simultaneously target human macrophage‐expressed membrane TNF‐α (hmTNF‐α) and IL‐23. Additionally, we fused the constant region of human IgG1 Fc (hIgG1 Fc) to BsNb to create BsNb‐Fc.  Our study encompassed in vitro and in vivo characterization of BsNb and BsNb‐Fc.

**Results:**

BsNb‐Fc exhibited an improved serum half‐life, targeting capability and effector function than BsNb. It's demonstrated that BsNb‐Fc exhibited superior anti‐inflammatory effects compared to the anti‐TNF‐α mAb (infliximab, IFX) combined with anti‐IL‐12/IL‐23p40 mAb (ustekinumab, UST) by Transwell co‐culture assays. Notably, in murine models of acute colitis brought on by 2,4,6‐trinitrobenzene sulfonic acid（TNBS) and dextran sulphate sodium (DSS), BsNb‐Fc effectively alleviated colitis severity. Additionally, BsNb‐Fc outperformed the IFX&UST combination in TNBS‐induced colitis, significantly reducing colon inflammation in mice with colitis produced by TNBS and DSS.

**Conclusion:**

These findings highlight an enhanced efficacy and improved biostability of BsNb‐Fc, suggesting its potential as a promising therapeutic option for IBD patients with insufficient response to TNF‐α inhibition.

**Key points:**

1. A bispecific nanobody (BsNb) was created to target TNF‐α and IL‐23p19, exhibiting high affinity and remarkable stability.
2. BsNb‐Fc inhibited the release of cytokines in CD4+T cells during co‐culture experiments.
3. BsNb‐Fc effectively alleviated colitis severity in mouse model with acute colitis induced by DSS or TNBS, outperforming the IFX&UST combination.

## Graphical abstract

Schematic illustration of engineering a bispecific nanobody‐based nanotherapy for targeted treatment of inflammatory bowel disease (IBD). (A) Development of the anti‐TNF‐α & IL‐23 bispecific nanobody (BsNb) and its quadrivalent structure (BsNb‐Fc). The BsNb and BsNb‐Fc were constructed and screened, demonstrating high affinity and stability. (B) Mechanism of targeted therapy of colitis by BsNb‐Fc. The nanocarrier of BsNb‐Fc exhibits intrinsic adhesive capability and penetrates the destructed intestinal barrier to target the inflamed colon. BsNb‐Fc binds to the membrane‐bound TNF‐α (mTNF‐α) and IL‐23 secreted by macrophages, effectively blocking the TNF/TNFR and IL‐23/IL23R cell signaling pathways in TNFR2^+^IL23R^+^CD4^+^ T cells. This disruption leads to the suppression of CD4^+^ T cell proliferation and differentiation and induces apoptosis, thereby alleviating intestinal mucosal inflammation. The therapeutic strategy shows promise for IBD patients unresponsive to anti‐TNF‐α therapy.

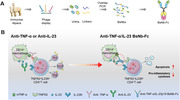

## 1 INTRODUCTION

Despite significant advancements in medical therapeutic approaches, such as corticosteroids, immunosuppressants and anti‐TNF‐α monoclonal antibodies (mAbs), existing therapies for inflammatory bowel disease (IBD) still face limitations in achieving satisfactory response and remission rates.^1^ Anti‐TNF‐α mAb, such as infliximab (IFX) and adalimumab (ADA), are commonly employed for IBD patients unresponsive to conventional therapy.^2^, ^3^ These mAbs target membrane‐bound TNF‐α (mTNF‐α) on CD14^+^ macrophages and T cells in the intestinal mucosa, impeding the mTNF‐α‐TNFR2 co‐stimulatory pathway in CD4^+^ T cells, which results in apoptosis induction, suppression of inflammatory signal amplification and mitigation of intestinal mucosal damage instigated by inflammatory cytokines. However, a significant proportion of Crohn's disease (CD) patients exhibit inadequate response to anti‐TNF‐α therapy, and a substantial percentage experiences secondary loss of response.^4^, ^5^, ^6^, ^7^, ^8^, ^9^

In patients refractory to anti‐TNF‐α treatment, a distinct subset of TNFR2^+^IL23R^+^CD4^+^ T cells with heightened IL‐23 receptor expression has been implicated in anti‐TNF‐α treatment molecular resistance in CD.^10^ Notably, ustekinumab (UST), an antibody that targets the p40 subunit shared by IL‐12 and IL‐23, has shown high rates of clinical remission in CD patients’ refractory or intolerant to anti‐TNF‐α therapy.^11^, ^12^, ^13^, ^14^ Specific inhibitors of IL‐23p19, such as risankizumab and brazikumab, have also shown remarkable efficacy in IBD patients refractory to anti‐TNF‐α.^15^, ^16^ Our team recently conducted a preliminary study to review and categorize clinically available dual‐target combinations of mAbs based on their mechanisms of action. We identified three major categories: overlapping effects, synergistic effects, and complementary effects. Among these combinations, we concluded that synergistic effects exist between anti‐IL‐12/23p40 and anti‐TNF‐α agents.^17^ The therapeutic use of UST and anti‐TNF‐α antibodies appears to be safe and well‐tolerated in IBD patients, although the number of patients involved in the study is limited.^18^, ^19^, ^20^

Using bispecific antibodies (BsAbs) to target TNF‐α and IL‐23p19 simultaneously has emerged as a promising strategy to improve therapeutic efficacy and simplify combinatorial therapy for IBD.^21^, ^22^ BsAbs has primarily been used in oncology,^23^, ^24^, ^25^, ^26^ recent research has demonstrated that the bispecific antibody Amivantamab exhibits long‐lasting and sustained remission in the management of non‐small cell lung cancer (NSCLC) with cEGFR mutations, marking a significant breakthrough in cEGFR‐mutated NSCLC therapy.^27^ However, the potential of BsAb in IBD clinical applications to enhance therapeutic efficacy is foreseeable. In some autoimmune diseases, BsAbs play a therapeutic role in preventing immune contacts by neutralizing inflammatory cytokines or focusing on surface indicators between activated T cells and B lymphocytes or antigenic presenting cells.^28^

BsAbs are currently categorized into three major structural classes: antibody fragments, or alternative scaffold proteins fused to an immunoglobulin Fc region or to proteins that increase their pharmacokinetic properties and valency (e.g. DVD‐Igs and IgG‐scFvs), and recombinant IgG BsAbs that maintain the structure of native IgGs.^29^ Among these options, BsAbs consisting of fusions of Fc and nanobodies offer the advantage of multivalency with reduced mass.^30^, ^31^ Nanobodies, which preserve the entire binding capacity in a single, stable domain, provide design flexibility and allow the creation of immunoreagents with structures tailored for specific applications.^32^, ^33^, ^34^ Fusion with Fc domains modifies their clearance kinetics, avidity and receptor‐mediated effector functions, leading to a significant increase in therapeutic response.^35^ Thus, BsNb‐Fc, which combines nanobodies and Fc domains, presents a promising avenue for IBD therapy, capitalizing on the structural advantages of nanobodies in terms of design flexibility and therapeutic response. However, comprehensive studies are still necessary to fully evaluate the potential of these molecules in clinical settings.

In this study, we have developed a bispecific nanobody (BsNb) capable of simultaneously neutralizing the bioactivities of hIL‐23p19 subunit and hTNF‐α. To enhance their affinity and thermal stability, we further structured BsNb‐Fc by fusing the constant region of human IgG1 Fc (hIgG1 Fc). In vitro binding analysis revealed that the binding affinity of BsNb‐Fc to TNF‐α and IL‐23 was significantly enhanced compared with BsNb. Using a dextran sulphate sodium (DSS)‐induced mouse colitis model, we observed that BsNb‐Fc exhibited greater potency than anti‐TNF‐α mAb IFX and effectively alleviated intestinal mucosal inflammation. To compare the efficacy of BsNb‐Fc with the combination of IFX and UST, we evaluated their effects on cell proliferation and cytokine secretion in as well as in Transwell co‐culture experiments involving macrophage RAW264.7 and THP‐1 cells, respectively, and macrophages and CD4^+^ T cells. The results demonstrated that BsNb‐Fc exerted a regulatory effect on macrophage cytokine secretion, thereby restraining T‐cell differentiation. The regulatory effect of BsNb‐Fc was superior to that of the IFX&UST combination. We evaluated the effect of BsNb‐Fc on the cytokine production of CD4^+^ T cells in a co‐culture system with THP‐1 cell culture supernatant. The findings showed that BsNb‐Fc might inhibit the release of IL‐17A, TNF‐α and IFN‐γ. Furthermore, in a TNBS‐induced mouse colitis model, BsNb‐Fc induced a more effective remission of mucosal inflammation compared to the combination of IFX&UST. Our study highlights that BsNb‐Fc effectively blocks the TNF/TNFR and IL‐17/IL‐23 axes in the intestinal, preventing immune over‐activation and resistance against TNF therapy mediated by TNFR2^+^IL23R^+^CD4^+^ T cells. This therapeutic approach reduces the risk of adverse reactions associated with ‘sequential administration’ and provides a promising new strategy for IBD patients.

## 2 RESULTS

### 2.1 Formatting, expression and purification of BsNbs

To generate BsNbs targeting both TNF‐α and IL‐23, we utilized four previously characterized nanobodies from two studies.^36^, ^37^ VHH#1 and VHH#2 have been shown to have affinities of 5.31 and 4.42 nM, respectively, for TNF‐α. On the other hand, VHH#22 and VHH#37 bind to the IL‐23p40 subunit and IL‐23p19 subunit, with affinities of 7.25 and 6.17 nM, respectively. These nanobodies were successfully expressed in *E. coli TransB*, and their binding specificity was confirmed using ELISA (Figure S1).

**FIGURE 1:**
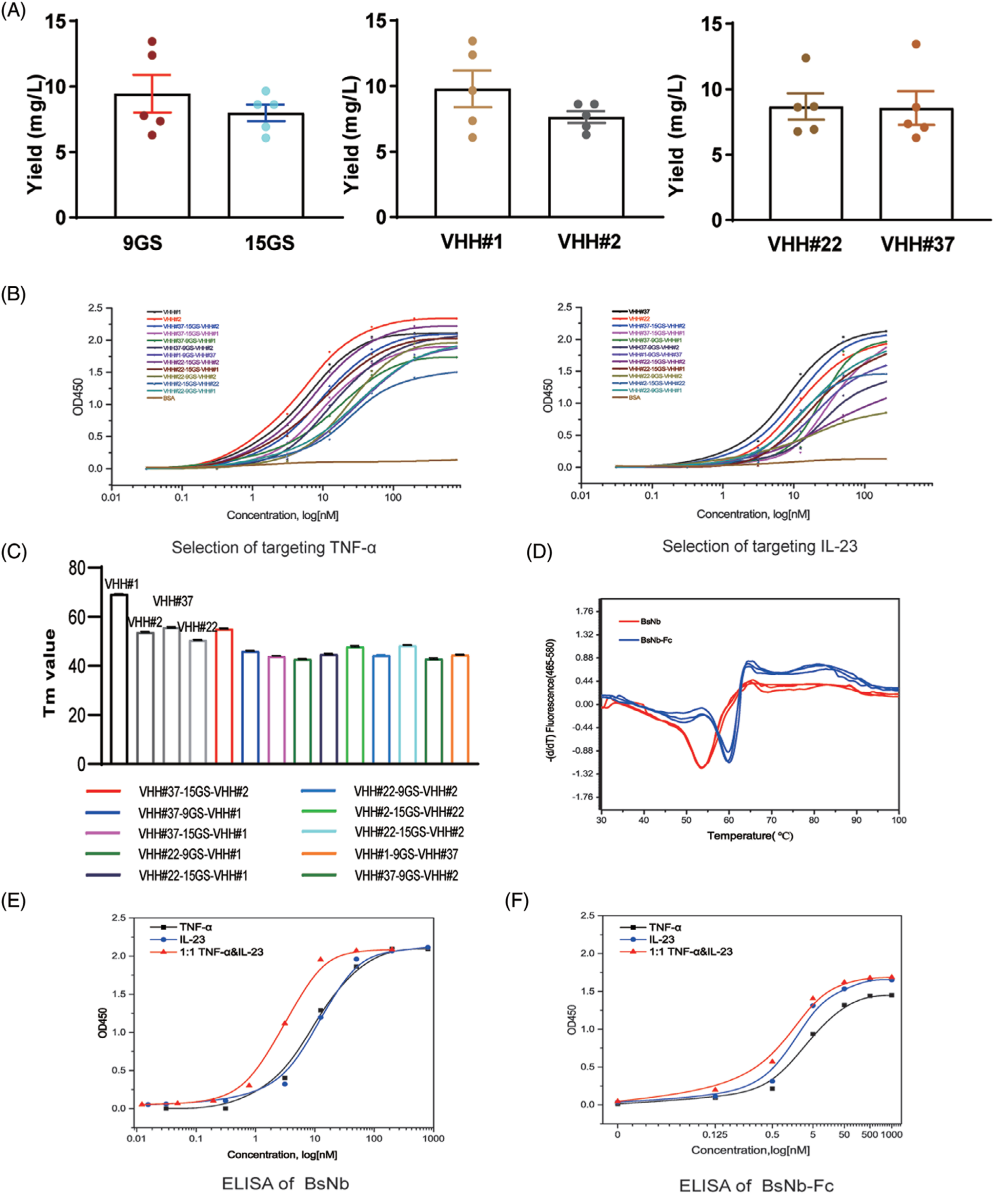
Expression, screen and characterization of BsNbs. (A) The yield of BsNbs was evaluated by assessing the influence of three components (VHHs and Linkers) used for their construction. (B) The binding efficiency of the BsNbs against two targets, TNF‐α and IL‐23, respectively, was measured by ELISA. The data represent the mean values of three independent experiments. (C) Thermal stability of BsNbs by measuring Tm values using differential scanning fluorimetry. (D) Temperature‐dependent melting curves of VHH#37‐15GS‐VHH#2 (red line) and BsNb‐Fc (blue line). Three independent measurements were conducted for each sample. (E and F) Binding of VHH#37‐15GS‐VHH#2 and VHH#37‐15GS‐VHH#2‐Fc to 100 nM TNF‐α, 100 nM IL‐23 and 1:1 mixture of 100 nM TNF‐α and IL‐23, respectively, was evaluated in a dose‐dependent manner.

For the construction of BsNbs, we linked a nanobody specific for TNF‐α with a nanobody specific for IL‐23, resulting in a total of 13 bispecific constructs (Table S3). To facilitate affinity purification, a His‐tag was added to the C‐terminus of the nanobodies (Figure S2). Out of the 13 constructs, 10 were purified as soluble proteins and eluted according to their expected mass in gel filtration. However, the remaining three constructs formed inclusion bodies and were not used for further experiments. Notably, we observed a decrease in solubility when VHH#2 was positioned at the N‐terminal (Figure 1A).

### 2.2 Evaluation of BsNb and BsNb‐Fc

The binding capacity and thermal stability of the BsNbs were assessed through indirect ELISA, surface plasmon resonance (SPR) and differential scanning fluorimetry. Results obtained from the ELISA test revealed that most BsNbs exhibited lower binding capacity towards both targets compared to the corresponding single‐domain VHHs (Figure 1B). The thermal melting (Tm) values of the BsNbs were lower than those of the original nanobodies (Figure 1C). Among the 10 BsNbs evaluated, the construct VHH#37‐15GS‐VHH#2, with desirable yields, apparent affinity and thermal stability, was selected for further experiments. To enhance stability and in vivo half‐life, the construct was fused to the human IgG1 Fc domain, resulting in the chimeric tetravalent molecule BsNb‐Fc (Figure S2). BsNb‐Fc was expressed in HEK293 cells and purified as a dimer by protein A affinity at a concentration of 50 mg/L. The thermal stability of BsNb‐Fc (60.05°C) was significantly higher than that of BsNb (55.13°C, Figure 1D). The simultaneous binding ability of BsNb‐Fc to both targets was assessed using ELISA, comparing its apparent affinity in the presence of single targets or their 1:1 molar mix.^38^ The presence of both targets resulted in an evident avidity effect, with a fivefold increase in binding for BsNb (Figure 1E). Although less pronounced for BsNb‐Fc, the apparent absolute binding capacity was already substantially elevated (.8 nM, Figure 1F).

**FIGURE 2:**
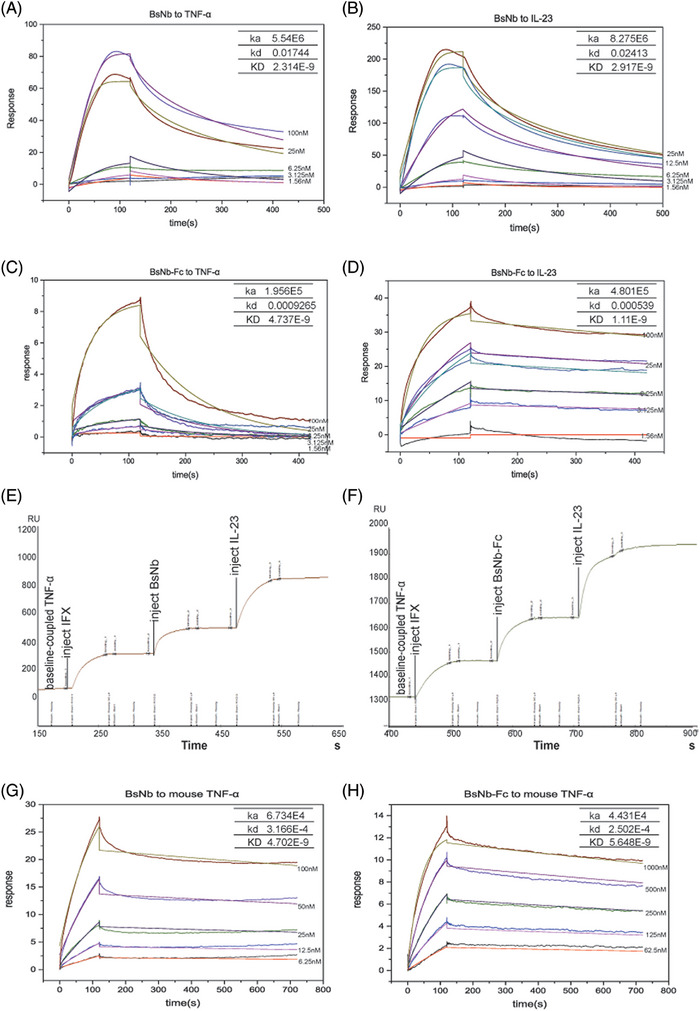
Binding affinity analyses of BsNb and BsNb‐Fc. (A and B) Affinity testing of the VHH#37‐15GS‐VHH#2 targeting TNF‐α and IL‐23 individually. Each coloured line represents a different nanobody concentration. (C and D) Affinity testing of VHH#37‐15GS‐VHH#2‐Fc targeting TNF‐α and IL‐23 individually. (E and F) Epitope binning assay of VHH#37‐15GS‐VHH#2 and VHH#37‐15GS‐VHH#2‐Fc performed with the approved infliximab. The curves include four processes: baseline, TNF‐α coupling; injection of infliximab (binding and dissociation); injecting BsNb (E) and BsNb‐Fc (F) (binding and dissociation); injection of IL‐23 (binding and dissociation). (G and H) Affinity testing of VHH#37‐15GS‐VHH#2‐Fc targeting mouse TNF‐α.

More precise affinity values were determined through SPR measurements (Figure 2). The results indicate that the BsNb exhibits affinities of 2.314 and 2.917 nM for TNF‐α and IL‐23, respectively, while the BsNb‐Fc demonstrates affinities of 4.737 and 1.11 nM for these two antigens. Detailed analysis of the binding constant (Ka) and dissociation constant (Kd) reveals that BsNb‐Fc displays reduced binding and dissociation rates, thus exhibiting a ‘slow association‐slow dissociation’ mode. As a control, IFX was employed, and its apparent affinity was determined to be .66 nM (Figure S3), which showed a binding pattern similar to the one constructed for BsNb‐Fc. To further investigate the binding capabilities of the bispecific constructs, a sequential injection experiment was performed. TNF‐α was initially used as the ligand, followed by the injection of IFX and either BsNb or BsNb‐Fc. The ability of the bispecific constructs to simultaneously bind both IL‐23 and TNF‐α at a different site than recognized by IFX was confirmed through experiments outlined in Figure 2E,F.

Furthermore, considering the validation of BsNbs in murine models, we assessed the binding affinity of BsNb and BsNb‐Fc towards mouse TNF‐α using SPR analysis. The results of the binding assays indicate that BsNb exhibited an affinity of 4.702 nM for mouse TNF‐α, whereas BsNb‐Fc demonstrated affinities of 5.648 nM. Detailed binding kinetics and outcomes are depicted in Figure 2G,H.

### 2.3 Effects of BsNbs on DSS‐induced colitis in mice

**FIGURE 3:**
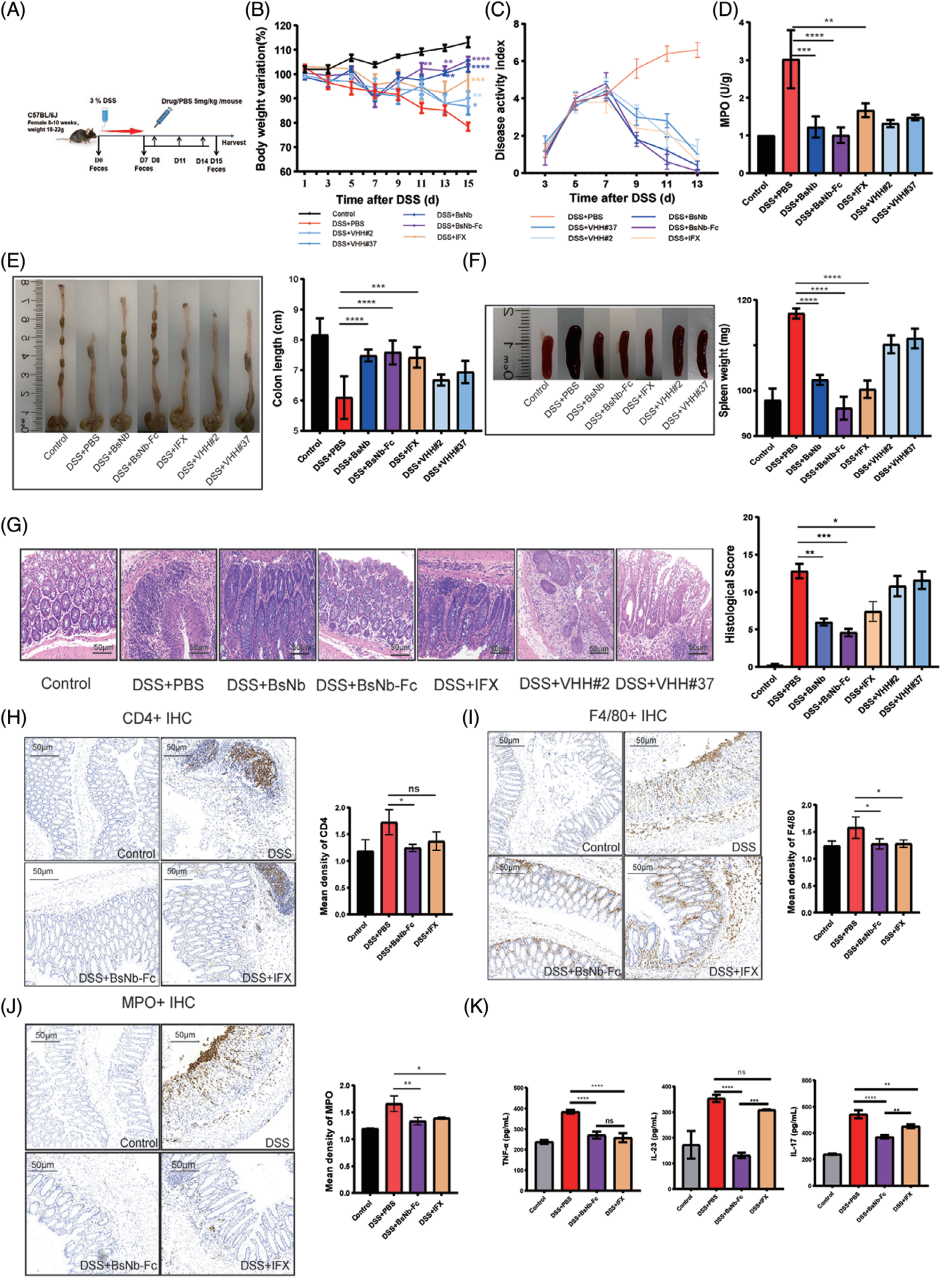
Amelioration of inflammatory symptoms in DSS‐induced colitis mice by antibody treatment. (A) Experimental protocol illustrating the induction of DSS‐induced colitis in mice. Mice (*n* = 5) were administered DSS (3%) in their drinking water for 7 days, followed by treatment with antibodies (BsNb, BsNb‐Fc, IFX, VHH#2 and VHH#37) at a dose of 5 mg kg^−1^ body weight for 7 days. (B) Body weight variation curves depicting the changes in body weight of mice treated with antibodies compared to the controls. (C) Disease activity index (DAI) scores of the different treatment groups over time, reflecting the severity of colitis. (D) Myeloperoxidase (MPO) activity in different treatment group, with MPO levels in the control group considered as the reference unit. (E and F) Evaluation of colon length and spleen weight in mice subjected to DSS and antibody treatment on day 15. Representative images and measurements of colon and spleen length and morphology were shown. (G) Representative histological images of colon tissues stained with H&E, with corresponding calculation of pathological score to assess colonic tissue damage. Scale bars, 50 µm. (H, I and J) Immunohistochemical (IHC) staining and quantitation of CD4^+^, F4/80^+^ and MPO^+^ in the proximal colon of controls, DSS‐treated, BsNb‐Fc and IFX‐treated mice. Scale bars, 50 µm. (K) Measurement of cytokine expression levels in sera of controls, DSS‐treated, BsNb‐Fc and IFX‐treated mice using ELISA. All data represented as mean ± SEMs; *p < .05; **p < .01; ***p < .001; ****p < .0001.

We created a mouse model of inflammatory bowel illness produced by DSS to evaluate the impact of BsNbs on the condition. Seven experimental groups were included in the study: a control group consisting of healthy mice, mice with colitis brought on by DSS and mice with colitis given VHH#2, VHH#37, BsNb, BsNb‐Fc and IFX, respectively. Administrations were performed on days 8, 11 and 14 of the experiment, and the faecal samples were collected on days 0, 7 and 15 to monitor changes (Figure 3A). Throughout the study, any phenotypic changes were carefully documented.

### 2.4 Effects of BsNbs on inducing colitis remission

Compared to the control group, DSS‐treated colitis mice gradually exhibited progressive apathetic behaviour, reduced food and water intake, and significant weight loss (10%, from 20.26 ± .96 g to 17.87 ± .33 g) during the initial experimental week (Figure 3B). By day 7, all DSS‐induced colitis mice presented varying degrees of naked or hidden haematochezia, and loose and watery stools, leading to a gradual increase in the disease activity index (DAI) reaching five points (Figure 3C). Treatment with antibodies (BsNb, BsNb‐Fc and IFX, respectively) markedly improved the physiological conditions of the mice, including mental state, activity level, food intake and water intake. In contrast, the DSS‐treated group experienced further weight loss and deterioration in the DAI index. On day 14 of the experiment, some mice exhibited severe weight loss, bloody stools and pronounced motor impairment. As per our pre‐established humane endpoints, these animals were immediately euthanized to alleviate their suffering and ensure their welfare. The BsNbs demonstrated a slightly superior remission effect compared to the monospecific nanobodies and the commercial antibody IFX. Notably, the BsNb‐Fc group exhibited the most efficient weight loss remission.

The administration of DSS caused hyperaemia, intestinal wall thickening and ulceration, which led to an increase in spleen weight and a decrease in colon length. Our observations revealed a significant reduction in colon length and the presence of bloody ulcerations and necrosis in the colon lumen of the DSS model group compared to the control group (Figure 3E). Treatment with BsNbs and IFX partially restored colon damage and demonstrated superior efficacy compared to VHH treatment. Similar trends were observed in the assessment of the spleen damage. The average spleen weight in the DSS reference group was 117.026 ± 1.105 mg, whereas the control group exhibited a weight of 97.892 ± 2.578 mg. Treatment with BsNb‐Fc resulted in complete remission (96.148 ± 2.538 mg, *p* < .05), while BsNb and IFX also displayed substantial effectiveness, whereas VHHs showed limited effects (Figure 3F).

To evaluate the inflammatory response, myeloperoxidase (MPO) activity was analysed using assay kits after sacrificing mice on day 15. Following DSS treatment, MPO levels increased three‐fold. However, administration of BsNb, BsNb‐Fc, VHHs and IFX effectively restored MPO activity close to baseline levels. Among these treatments, BsNb‐Fc demonstrated the highest efficiency in restoring MPO activity (Figure 3D).

### 2.5 Therapeutic potential of BsNb‐Fc in restoring colon integrity and modulating immune cell composition in DSS‐induced murine colitis

H&E staining was applied to the tissue slices from the colon for histopathological analysis (Figure 3G). The DSS group exhibited a pronounced acute inflammatory response, mucosal erosion, hyperaemia, severe crypt destruction in most glands, neutrophil infiltration and goblet cell loss(Figure 3G). However, treatment with BsNb‐Fc significantly reduced or completely abolished these pathological features. The protective activity of BsNb‐Fc was evident through the reduction of mucosal hyperaemia, a thinner layer of muscle, and a decrease in the infiltration of inflammatory cells into the colon tissue. The therapeutic effect of BsNb‐Fc was superior to that of BsNb, while VHHs and IFX provided only limited protection.

To evaluate the impact of bispecific antibody treatments on immune cell infiltration, immunohistochemistry (IHC) assays were performed to assess the proportions of CD4^+^ T helper cells, neutrophils and macrophages in the colon tissue of mice from each group. The frequencies of CD4^+^ T cells were significantly higher in the colonic tissue of DSS‐treated mice compared to those in the control group (*p* < .05). Treatment with BsNb‐Fc significantly reduced the number of CD4^+^ T cells (*p* < .05), whereas IFX treatment did not show a significant effect (*p* = .16) (Figure 3H). Similarly, the quantity of MPO‐ and F4/80‐positive cells was considerably elevated in the colonic tissues of DSS‐treated mice. Treatment with BsNb‐Fc significantly reduced the expression levels of CD4^+^, F4/80^+^ and MPO^+^ positive cells (*p* < .05), while IFX exhibited to inhibit F4/80‐ and MPO‐positive cell infiltration in the inflamed colon (Figure 3I,J).

### 2.6 Suppression of pro‐inflammatory signalling pathways by BsNb‐Fc outperforms IFX in DSS‐induced murine colitis

**FIGURE 4:**
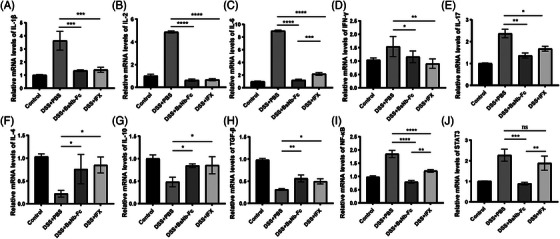
BsNb‐Fc treatment attenuates the overexpression of pro‐inflammatory cytokines induced by DSS in the colon tissue. The mRNA expression levels various pro‐inflammatory cytokines, including IL‐1β, IL‐2, IL‐6, IFN‐γ and IL‐17, as well as anti‐inflammatory cytokines IL‐4, IL‐10 and TGF‐β, were assessed. Additionally, the mRNA expression levels of NF‐κB and STAT3 were measured. Data are presented as the means ± SEM and were analysed using ordinary one‐way ANOVA with multiple comparisons. Statistical significance is denoted as *p < .05; **p < .01; ***p < .001; ****p < .0001, compared with the respective DSS+PBS group.

Pro‐inflammatory cytokines IL‐23, TNF‐α and IL‐17 were significantly elevated in the sera of DSS‐treated mice compared to control animals. Treatment with BsNb‐Fc and IFX significantly reduced the expression of these cytokines (Figure 3K). Subsequently, we assessed the mRNA expression of these cytokines in the DSS‐challenged colon tissues following treatment with BsNb‐Fc or IFX (Figure 4). Pro‐inflammatory cytokines IL‐1β, IL‐2, IL‐6 and IFN‐γ messenger RNA levels were significantly decreased by both treatments, while the expression of anti‐inflammatory cytokines IL‐4, IL‐10 and TGF‐β was enhanced. Notably, BsNb‐Fc exhibited superior efficacy compared to IFX in downregulating the expression of NF‐κB and signal transductor and activator of transcription 3 (STAT3), two critical transcriptional controllers of cytokines that promote inflammation and inflammatory response mediators. The colon tissue of mice treated with DSS exhibited a notable elevation of NF‐κB expression, as demonstrated by real‐time PCR analysis, which was completely suppressed by BsNb‐Fc and only partially by IFX treatment (Figure 4I). Our experimental findings confirmed a 2.272 ± .167‐fold increase in STAT3 mRNA in DSS‐induced colitis mice. Treatment with BsNb‐Fc significantly inhibited STAT3 expression (Figure 4J).

Additionally, in the DSS‐induced murine colitis model, we compared the effects of BsNb‐Fc with anti‐mouse TNF‐α mAb. The findings revealed that both BsNb‐Fc and anti‐mouse TNF‐α mAb ameliorated the DSS‐induced reduction in mouse body weight. However, BsNb‐Fc exhibited an earlier onset of efficacy compared to the anti‐mouse TNF‐α mAb group and ultimately achieved a superior alleviation rate by day 15 (Figure S5A). Histological evaluation of colonic tissues via H&E staining indicated significantly lower histopathological scores in the BsNb‐Fc group compared to the anti‐mouse TNF‐α mAb group, showcasing a better mitigation of DSS‐induced tissue damage (Figure S6). Furthermore, we assessed the mRNA expression of various cytokines in colonic tissues, demonstrating that BsNb‐Fc significantly reduced the expression of pro‐inflammatory cytokines, particularly exhibiting more pronounced modulation of IL‐23, IL‐17, IL‐1β and IL‐10 compared to the anti‐mouse TNF‐α mAb group (Figure S5).

### 2.7 Modulation of inflammatory cytokines and T‐cell responses by BsNbs under macrophage‐mediated inflammatory conditions

**FIGURE 5:**
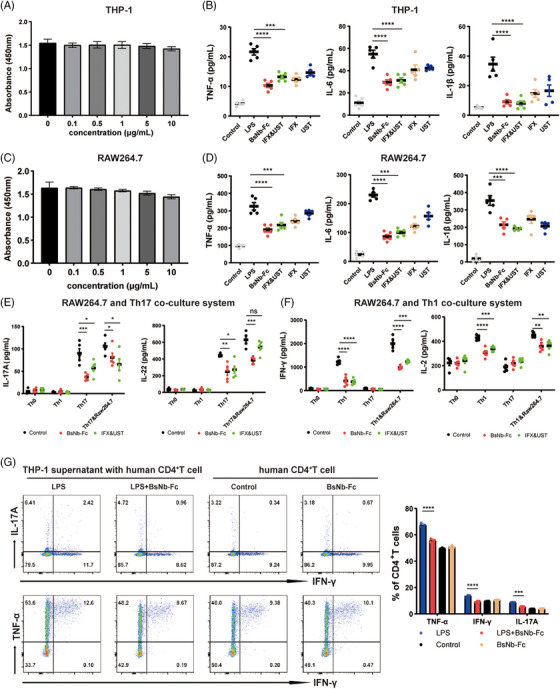
BsNb‐Fc modulates cytokine secretion in macrophages, resulting in downregulation of CD4^+^ T cell proliferation and differentiation. (A) Absorbance at OD450 nm of THP‐1 cells stimulated with 1 µg/mL LPS and treated with different concentrations of BsNb‐Fc (10, 5, 1, .5, .1 µg/mL). (B) ELISA measurement of TNF‐α, IL‐6 and IL‐1β cytokine levels in the medium of THP‐1 cells stimulated with 1 µg/mL LPS and treated with 10 µg/mL antibodies. (C) Absorbance at OD450 nm of RAW264.7 cells stimulated with .1 µg/mL LPS and treated with different concentrations of BsNb‐Fc (10, 5, 1, .5, .1 µg/mL). (D) ELISA measurement of TNF‐α, IL‐6 and IL‐1β cytokine levels in the medium of THP‐1 cells stimulated with .1 µg mL‐1 LPS and treated with 10 µg/mL antibodies. (E) Measurement of IL‐17A and IL‐22 levels in the co‐culture system of RAW264.7 and Th17 cells, where macrophages were stimulated with .1 µg/mL LPS and treated with 10 µg/mL antibodies. (F) Measurement of IFN‐γ and IL‐2 levels in the co‐culture system of RAW264.7 and Th1 cells, where macrophages were stimulated with .1 µg/mL LPS and 10 µg/mL antibodies. (G) Flow cytometry was used to evaluate the intracellular levels of TNF‐α, IFN‐γ and IL‐17A in a co‐culture of THP‐1 cell culture supernatant and human CD4^+^ T cells. Data are presented as the means ± SEM. Statistical significance: *p < .05; **p < .01; ***p < .001; ****p < .0001.

To assess the impact of BsNbs on inflammatory cytokine secretion by macrophages, we conducted In vitro experiments using mouse RAW264.7 and human THP‐1 macrophage cell lines. The stimulatory effects of lipopolysaccharide (LPS) on RAW264.7 and THP‐1 were determined in a preliminary experiment, revealing that RAW264.7 cells were stimulated by .1 µg/mL LPS and THP‐1 cells by 1 µg/mL LPS (Figure S4). The effects of different concentrations of antibodies (10, 5, 1, .5 and .1 µg/mL) on THP‐1 cell proliferation were evaluated by measuring absorbance at 450 nm using CCK8. Results demonstrated that BsNb‐Fc did not affect THP‐1 cell viability or number at these concentrations (Figure 5A). Similar findings were observed in RAW264.7 cells (Figure 5C). Furthermore, the levels of TNF‐α, IL‐6 and IL‐1β in the medium were measured to assess the impact of BsNb‐Fc on cytokine secretion by RAW264.7 cells induced by LPS. Treatment with BsNb‐Fc resulted in a decrease of these overexpressed cytokines, reducing their levels from 325.518 ± 21.544 pg/mL, 229.800 ± 7.611 pg/mL and 354.377 ± 27.464 pg/mL to 190.056 ± 12.436 pg/mL, 86.160 ± 7.217 pg/mL and 214.254 ± 17.185 pg/mL, respectively. Both BsNb‐Fc and IFX&UST demonstrated effective inhibition of macrophage inflammatory factor secretion (Figure 5D, *p* < .0001).

Following stimulation with relevant cytokines and antibodies, T cells differentiate into Th0 cells or Th17 cells. Compared to Th0 cells and Th1 cells, Th17 cells exhibited an increase in IL‐17A and IL‐22 secretion, with levels of 90.067 ± 10.348 pg/mL and 442.633 ± 44.787 pg/mL, respectively. Treatment with BsNb‐Fc and IFX&UST led to a decrease in IL‐17A expression to 37.061 ± 3.474 pg/mL and 57.111 ± 7.471 pg/mL, respectively, and IL‐22 expression to 246.758 ± 44.787 pg/mL and 271.300 ± 44.573 pg/mL, respectively. These findings indicate that BsNb‐Fc and IFX&UST effectively inhibit the differentiation of Th0 cells into Th17 cells.

In the co‐culture system, the secretion levels of IL‐17A and IL‐22 by Th17 cells in the absence of antibody intervention were 105.861 ± 7.488 pg/mL and 629.108 ± 38.022 pg/mL, respectively, suggesting that macrophages in the co‐culture system play a role in stimulating and differentiating Th17 cells. Treatment with BsNb‐Fc resulted in reduced expression of IL‐17A to 80.939 ± 9.682 pg/mL, and IL‐22 to 246.758 ± 44.787 pg/mL, respectively, while IFX&UST had less significant effects on cytokine secretion compared to BsNb‐Fc (Figure 5E). Both BsNb‐Fc and IFX&UST significantly reduced IFN‐γ (*p* < .001) and IL‐2 (*p* < .01) secretion by Th1 cells (Figure 5F). In summary, these results indicate that BsNb‐Fc regulates CD4^+^ T cell proliferation and differentiation by modulating cell proliferation activity and cytokine secretion during macrophage maturation. We conducted a co‐culture experiment using the human macrophage cell line THP‐1 and human CD4^+^ T cells to study the effects of BsNb‐Fc. By adding THP‐1 cell culture supernatant, which was stimulated with LPS and treated with BsNb‐Fc, to human CD4^+^ T cell culture and analysing cytokine secretion through flow cytometry, we discovered that BsNb‐Fc effectively suppressed the secretion of IL‐17A, TNF‐α and IF‐γ in the CD4^+^ T cell co‐culture system (Figure 5G).

### 2.8 BsNb‐Fc outperforms IFX&UST combination in ameliorating TNBS‐induced murine colitis

To compare the therapeutic effects of the BsNb‐Fc and IFX&UST combination in vivo, another mouse colitis model was established using TNBS. IFX was administered via intraperitoneal injection at a dose of 5 mg/kg body weight,^39^, ^40^ and UST was administered at a concentration of .5 mg/kg body weight.^41^ The dosage of mAbs in the combination therapy was halved to mitigate potential side effects associated with the Fc domain in antibody combination therapy. On day 7, the control group exhibited a growth rate of 117.230% ± 1.633 in body weight. In the TNBS group, mice experienced weight loss throughout an induction period, with a bodyweight variation of 81.594% ± 1.358 on day 7.

**FIGURE 6:**
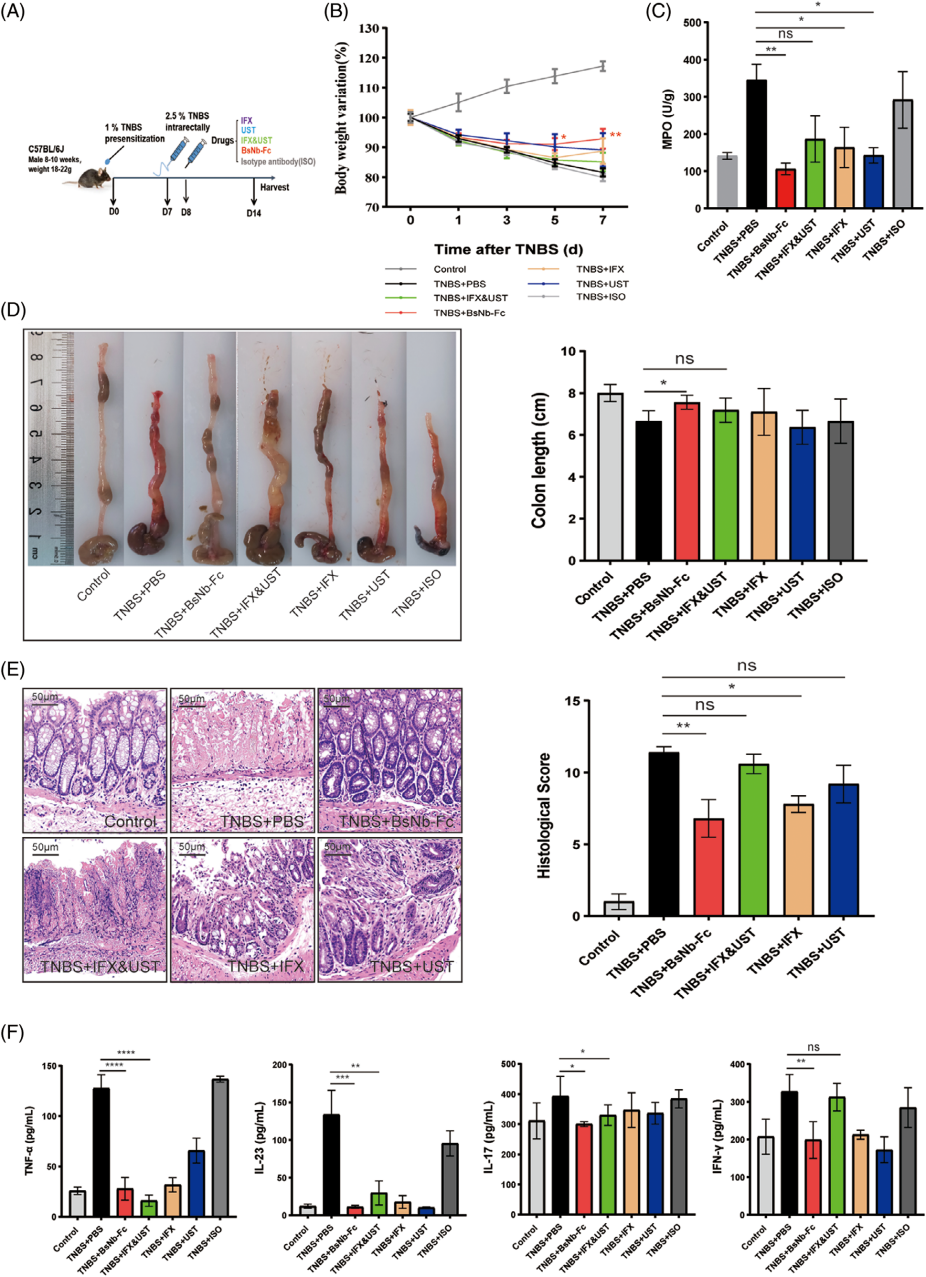
BsNb‐Fc demonstrates superior efficacy compared to IFX, UST and IFX&UST combination in a TNBS‐induced mouse colitis model. (A) Experimental protocol for TNBS‐induced colitis in mice. Mice (*n* = 5) were sensitized with TNBS (1%) on the abdominal skin for 7 days, followed by intrarectal administration of 2.5% TNBS. Subsequently, the mice were treated with the following antibodies: BsNb‐Fc (5 mg/kg), IFX (2.5 mg/kg) &UST (.25 mg/kg), IFX (5 mg/kg), UST (.5 mg/kg) and isotype antibody for 7 days. (B) Body weight variation of antibodies‐treated mice compared to controls. (C) MPO activity of different treatment groups using an MPO activity assay kit. (D) Assessment of colonic morphology and length in mice treated with TNBS and antibodies. (E) Representative histological images of colon tissues stained with H&E, and calculation of the pathological score of colonic tissues. Scale bars, 50 µm. (F) Measurement of cytokine expression levels in colon tissues using ELISA. All data represented as mean ± SEMs. Statistical significance: *p < .05; **p < .01; ***p < .001; ****p < .0001.

Treatment with BsNb‐Fc significantly mitigated TNBS‐induced weight loss on day 5 (*p* < .05). On day 7, the body weight variation was 92.889% ± 3.288 (*p* < .01) in the BsNb‐Fc‐treated mice, and the body weight remained higher than that of the colitis group throughout the induction and treatment period. Treatment with IFX and UST also provided relief in colitis, with a change rate in body weight of 88.751% ± 4.321 and 89.066% ± 5.620 on day 7, respectively. Surprisingly, the remission efficiency of the IFX&UST group was lower than that of mAb alone (Figure 6B), and two mice even died during treatment. Similar trends were observed in MPO expression in the colon tissue (Figure 6C).

The BsNb‐Fc treatment group exhibited the best performance. TNBS induction in mouse colons resulted in several pathological changes, including colonic mucosal ulcers, thickening of the colonic wall, loss of elasticity and colon shortening. Treatment with various antibodies showed varying degrees of relief in colon pathology, with the BsNb‐Fc group demonstrating the highest degree of alleviation. In contrast, the IFX&UST group exhibited noticeable abscess formation in the colonic tissue (Figure 6D). Subsequent examination of colon tissue through slicing and H&E staining revealed that BsNb‐Fc significantly mitigated TNBS‐induced mucosal ulcers, inflammatory infiltration and distorted glandular structures. Conversely, the IFX&UST group displayed pronounced inflammatory reactions, characterized by mucosal erosion and congestion, extensive crypt damage, neutrophil infiltration and loss of goblet cells, resulting in even more severe pathological changes compared to the TNBS group (Figure 6E). ELISA analysis of cytokines in the colon tissues (Figure 6F) demonstrated a significant increase in the expression levels of TNF‐α, IL‐23, IL‐17 and IFN‐γ in mouse colon tissue following a TNBS induction, measuring 127.453 ± 7.784 pg/mL, 133.802 ± 32.164 pg/mL, 392.846 ± 65.290 pg/mL and 327.564 ± 25.802 pg/mL, respectively. These cytokines are pro‐inflammatory factors closely associated with the development of IBD. Treatment with BsNb‐Fc led to a significant decrease in these pro‐inflammatory cytokines to 27.671 ± 6.468 pg/mL, 11.185 ± 2.051 pg/mL, 300.538 ± 7.697 pg/mL and 198.974 ± 28.121 pg/mL, respectively, indicating a notable decrease in their expression. In the IFX&UST treatment group, the levels of target cytokines TNF‐α and IL‐23 were reduced to 15.987 ± 3.229 pg/mL and 29.660 ± 16.109 pg/mL, respectively. However, the expression of IFN‐γ was higher than that of the TNBS group, measuring 312.513 ± 20.925 pg/mL.

## 3 DISCUSSION

Nanobodies have emerged as a promising alternative to conventional mAbs in various fields due to their unique structural features, making them amenable to modification and customization for specific purposes.^42^ Recently, progress in experimental studies and clinical applications of multivalent and multi‐specific nanobodies has highlighted their vast potential in addressing diverse challenges.^43^, ^44^, ^45^ Additionally, the development of multi‐paratopic nanobody‐based constructs as effective neutralizing drugs against SARS‐CoV‐2 has demonstrated the versatility and clinical utility of this approach.^46^, ^47^, ^48^ Given these advancements, we explored the possibility of applying this strategy to provide a novel therapeutic opportunity for IBD. In our study, we successfully developed a series of BsNbs without the Fc region, resulting in a reduced molecular weight of 32 kDa. This design facilitated better tissue penetration compared to IgG antibodies, enhancing in vivo antigen targeting. We optimized the linker length and reciprocal position of the two nanobodies at the N‐terminal and C‐terminal to ensure stability and functionality.^49^, ^50^ Furthermore, the rational design of the structure holds the potential for further improving the present BsNbs, leveraging their monomeric nature and short sequence for in silico modelling and computational enhancements of their biophysical features, as well as humanization.^32^, ^51^, ^52^, ^53^

To enhance molecule stability and circulation half‐life, we incorporated an Fc domain into the BsNbs. This modification resulted in an increased yield and prolonged in vivo circulation half‐time, thereby potentially enhancing the therapeutic effects, as demonstrated in our experimental colitis model in mice (Figure 3). Additionally, the Fc receptor (FcR)‐mediated activity of the Fc domain significantly contributes to the therapeutic response of anti‐TNF‐α antibodies in IBD. This has been observed in the case of anti‐TNF‐α mAbs like IFX and adamuzumab, which have shown the ability to induce complete mucosal healing in CD patients, whereas the anti‐TNF F(ab)2 fragment (cetuzumab) has exhibited a limited efficacy.^54^ Furthermore, there exists interindividual variability during mAb therapy. Studies have discussed the immunogenicity of different anti‐TNF‐α mAbs, IFX and ADA, investigating the critical function of immune complexes − rather than FcRn − in mouse immunization against anti‐TNF‐α antibodies. Separate injections of IFX, ADA or IFX pre‐incubated with TNF‐α were given to wild‐type and FcRn knockout mice. IFX showed species specificity, but ADA showed cross‐reactivity with murine TNF‐α. Only ADA, when given by itself, produced a humoral response. TNF‐α‐induced immune complexes induced an immunological response to IFX.^55^ Notably, Qiu et al. demonstrated that treating mice with IFX suppressed p53‐upregulated modulator of apoptosis (PUMA) expression and colitis induced by DSS and TNBS.^56^ Ninnemann et al. used a mouse model known as human TNF knock‐in (hTNF‐KI), in which the murine equivalent of the TNF gene was substituted with the human gene and its promoter. Using naive T cells extracted from hTNF‐KI donor animals, they created a mouse colitis model that was exclusively dependent on human TNF expression in the progeny of hTNF‐KI mice bred with Rag1^−^/^−^ mice.^57^ These studies underscore that a variety of particular anti‐human TNF medications with unique pharmacological and physicochemical properties are used in clinical treatment; these compounds cannot be completely evaluated in the mouse models that are now available. Our BsNb exhibits cross‐reactivity between human and murine sources.

Combining therapies has emerged as a solution for the complexity of IBD treatment. Single therapies often fall short in a heterogeneous disease landscape, resulting in clinical remission failures and loss of treatment response.^58^ Combining drugs that target multiple biological pathways has emerged as a recent approach to overcome these challenges and prevent resistance,^59^ such as anti‐TNF‐α and anti‐IL‐23 antibodies, offering potential synergistic effects.^60^ Some studies have reported positive outcomes with UST plus IFX,^61^, ^62^ UST plus ADA,^63^ leading to clinical responses and endoscopic improvements, though adverse events were observed in some cases. However, this approach is not without drawbacks. It is associated with an increased risk of serious adverse events like infections, similar to combined therapies for conditions like rheumatoid arthritis. Retrospective studies have shown promise in patients with CD or ulcerative colitis (UC) receiving combination therapy involving anti‐TNF and UST, resulting in clinical responses without adverse reactions.^64^

In summary, combined therapy shows promise in improving targeted outcomes but introduces additional safety considerations. Concurrent administration of different biologics often necessitates intricate dose adjustments for each treatment, requiring extensive dose‐range exploration studies. In our experimental research, the combination treatment of IFX and UST for TNBS‐induced colitis in mice did not yield the anticipated clinical remission efficiency and led to severe adverse effects. In clinical practice, a ‘sequential administration’ approach is frequently employed, involving the sequential administration of multiple targeted drugs to patients rather than simultaneous administration. In this context, the development of a single molecule with multiple activities, such as bispecific antibodies, has the potential to simplify dose assessment and potentially yield combined therapies with a more favourable benefit‐risk ratio. It is important to note that, in this study, the dosing regimen for the combination of mAbs was not extensively explored, to offer a mechanism‐based alternative therapeutic approach. To mitigate the risk of excessive immune responses induced by the Fc segment, we opted to halve the dosage of each mAb. This approach aims to strike a balance between therapeutic efficacy and safety considerations, providing a potential avenue for further investigation in the pursuit of optimized combination therapies.

Although no clinical trials based on BsAb have been initiated for IBD patients, several promising molecules are currently under development. For instance, AZ17 is a BsAb in preclinical stages that combines binders specific for IL‐23 and IL‐6, and it has demonstrated high efficacy in a human xenograft transplantation model of psoriasis.^65^

In patients who do not respond to anti‐TNF therapy, intestinal TNFR2^+^IL23R^+^CD4^+^ T cells appear to be activated by IL‐23 secreted from CD14^+^ macrophages, despite the blockade of the TNF‐TNFR signalling by TNF‐α antibodies.^66^ Since TNFR2^+^IL23R^+^ CD4^+^ T cells can still exert anti‐apoptotic effects through the IL‐23‐IL23R/STAT3 pathway, effective therapy necessitates the simultaneous blockade of both the TNF‐α and IL‐23 pathways. In our study, we described the process of designing and developing a novel BsNb (BsNb‐Fc) capable of simultaneously binding to TNF‐α and IL‐23p19. These findings propose a promising therapeutic avenue utilizing BsNbs to target the inflammatory cascade in immune diseases.

## 4 CONCLUSION

Our study demonstrates the superiority of BsNbs over the combination of anti‐TNF‐α and anti‐p40 mAbs in inducing clinical remission. The current scenario in IBD treatment involves different companies owning drugs suitable for targeted therapy, resulting in limited opportunities for clinical trials exploring drug combinations due to patent restrictions. However, our data strongly suggest that the BsNb constructs characterized in this study hold great potential as a novel therapeutic strategy for not only IBD but also other related conditions, such as rheumatoid arthritis and ankylosing spondylitis. These findings pave the way for further research and development in the field of immunotherapy for immune‐mediated diseases, offering promising prospects for improved patient outcomes.

## 5 MATERIALS AND METHODS

### 5.1 Anti‐TNF‐α and anti‐IL‐23 nanobodies

The anti‐TNF‐α nanobodies VHH#1 and VHH#2, as well as the anti‐IL23 nanobodies, VHH#22 and VHH#37, were previously described.^36^, ^37^

### 5.2 Generation of recombinant human TNF‐α and IL‐23 proteins

Recombinant human TNF‐α and IL‐23 proteins were produced in HEK293 cells (KaiRui Biotech) transformed with pcDNA3.1(+) vector. The HEK293 cells were cultured in KOP293 culture medium (KaiRui Biotech) at 37°C, 5% CO_2_ and 120 r/min. Cells were transfected when the cell density reached 2×10^6^/mL. The plasmid‐carrier complex was prepared using KPM and TA‐293 transfection reagents (KaiRui Biotech). After 24 h of culture, cell protein expression enhancer (KE 293) and nutritional additives (KT‐FEED 50×) were added to increase the protein yields. After 7 days of culture, cells were centrifuged for 15 min at 4000×*g*. Target His‐tagged proteins were purified from the resulting supernatants using 5 mL of Ni Sepharose High‐performance resin (Cytiva) and eluted with 300 mM imidazole. The final purified samples were desalted into phosphate buffer saline (PBS) buffer for later tests and separated on 10% SDS–PAGE.

### 5.3 Formatting, expression and purification of BsNbs

Bispecific constructs targeting TNF‐α and IL‐23 simultaneously were prepared by connecting two monovalent nanobody sequences using either a 9GS linker (Gly)4Ser (Gly)3Ser or a 15GS linker (Gly)4Ser (Gly)4Ser (Gly)4Ser obtained by overlap extension PCR. The constructs were inserted between the XbaI and XhoI of a pET‐32a (+) plasmid. *E. coli* TransB (DE3) was transformed using the resultant plasmid, and the transformed cells were cultured in an Luria‐Bertani（LB) medium enhanced with ampicillin. The parameters for the induction temperature (16°C and 20°C) and IPTG concentration (.1, .2, .5 and 1.0 mM), and induction time (8 and 16 h) were optimized. Bacteria were centrifuged for 10 min at 6000×*g*, then resuspended in PBS, sonicated for 20 min, and the lysate was finally centrifuged at 6500×*g*. The resulting supernatants were loaded onto a Ni Sepharose High‐performance resin (Cytiva) and the BsNbs were eluted with 300 mM imidazole.

Tetravalent bispecific nanobody (BsNb‐Fc) was obtained by fusing the BsNb sequence to that corresponding to the human IgG1 antibody's Fc domain. The BsNb‐Fc constructs were cloned into the pcDNA 3.1(+) vector and expressed as previously mentioned. The supernatants were purified using HiTrap ProteinA High‐performance resin (Cytiva). The constructs were eluted at acidic pH (50 mM acetate buffer, 20 mM NaCl, pH 3.5) and immediately neutralized with 1 M Tris‐HCl pH 8.8.

Both BsNbs and BsNb‐Fc samples were buffer‐exchanged in PBS using a 5 mL HiTrap Desalting column (Cytiva). The final products were filtered using a .22 µm syringe filter to achieve sterilization. Every buffer that was utilized lacked endotoxins.

### 5.4 Enzyme‐linked immunosorbent assay of BsNbs

The recombinant hTNF‐α and hIL‐23 were diluted in coating buffer (50 mM carbonate buffer, pH 9.6) to a concentration of 20 µg/mL. The diluted proteins were then applied to each well of a 96‐well plate, and the plate was incubated at 4°C for an entire night. As a negative control, bovine serum albumin was used. The following day, after three phosphate buffered saline with tween 20 (PBST) washes, the plate was blocked for 2 h at 37°C in PBS containing 5% milk. After additional washing in PBST, BsNbs at concentrations ranging from 3.125 to 200 µg/mL were put into the wells, and they were incubated for 2.5 h at 37°C. Following another round of washing, HRP‐labelled anti‐His mouse monoclonal secondary antibodies were added and incubated at 37°C for 2 h. The colour reaction was initiated by adding 50 µL of 3,3',5,5'‐Tetramethylbenzidine (TMB) substrate per well. Following a 10‐min dark incubation period, the reaction was terminated by adding 100 µL H_2_SO_4_ to each well, and the Varioskan LUX microplate reader (Thermo Scientific) was utilized to measure the absorbance at OD450.

### 5.5 Thermal stability measurements

Temperature‐dependent fluorescence variation was measured using an Instrument LightCycler 480 II (Roche). Twenty microlitres in total, of which 2 µL of BsNbs (20 µM), .04 µL of 250 mM TCEP and 1 µL of 200× SYPRO Orange dye (Sigma‐Aldrich) in PBS was prepared. The mixture was subjected to an excitation wavelength of 465 nm and an emission wavelength of 580 nm, with a temperature gradient between 25°C and 95°C. The acquisition mode was set to continuous with a rate of .01/s. The melting temperature (Tm) values were calculated using the LightCycler Thermal Shift Analysis software (Roche Applied Science) and Origin 8.0 software.

### 5.6 Surface plasmon resonance

A Biacore T200 (Cytiva) was used for SPR investigation. The running buffer used for all experiments was PBS. The recombinant proteins, hTNF‐α and hIL‐23, were amino‐immobilized to the CM5 chip (Cytiva). hTNF‐α was diluted in sodium acetate buffer (pH 5.0) to a concentration of 5 µg/mL, while hIL‐23 was diluted to the same concentration in the same buffer at pH 4.5. The immobilization levels obtained were 500 Ru for hTNF‐α and 800 Ru for hIL‐23. The biosensor surface was blocked with ethanolamine‐HCL (1.0 M). BsNb and BsNb‐Fc samples at concentrations ranging from 1.56 to 100 nM were injected over the chip at a 30 µL/min flow rate, with a 120‐s contact time and a 900 s dissociation period. The Biacore T200 Evaluation Software's 1:1 binding model was used to analyse the data (version 3.2.1) to determine the apparent affinity of the bivalent molecules. The binding between hTNF‐α and IFX was evaluated by injecting IFX samples at concentrations between 3.12 and 50 nM using the same conditions described previously.

To test the capacity of BsNb/BsNb‐Fc to bind to both targets simultaneously, a biosensor coated with either BsNb or BsNb‐Fc at 500 and 1000 Ru, respectively, was used. First, hTNF‐α (100 nM) was injected for 120 s. Once the binding reached a stable plateau, 100 nM of IFX was injected for 120 s, followed by the injection of 100 nM of IL‐23 for 120 s. For 30 s, the chip was regenerated using 10 mM glycine buffer (pH 2.0).

To assess the binding of BsNb/BsNb‐Fc with mouse TNF‐α, the mouse TNF‐α (UA Bioscience, UA040173) was amino‐immobilized onto the CM5 chip by diluting it to a 20 µg/mL concentration in pH 4.0 sodium acetate buffer. The immobility of the target levels achieved was 2000 RU. Samples of BsNb and BsNb‐Fc, ranging in concentrations from 6.25 to 100 nM and 62.5 to 1000 nM, respectively, were applied over the chip with a contact time of 120 s and a dissociation time of 900 s.

### 5.7 DSS‐induced colitis model in mice

Thirty‐five specific pathogen‐free (SPF), 18–22 g of female C57BL/6J mice were obtained from Beijing HFK Bio‐Technology and divided into seven groups at random: group under control (untreated), DSS model group, DSS+TNF‐α nanobody administration group, DSS+IL‐23p19 nanobody group, DSS+BsNb group, and DSS+BsNb‐Fc and DSS+IFX group. DSS (40 kDa, MP Biomedicals) was dissolved in sterile distilled water to prepare a 3% solution and was allowed ad libitum access to this solution for 7 days. Through sequence alignment, we observed a high degree of conservation in the cell factor sequences between human and murine sources. This guided our choice for the dosage of IFX antibody injections in our animal experiments, referencing previous literature on murine models.^67^ The antibody was diluted to a 100 µg/mL concentration in PBS and administered via intraperitoneal injections of 500 µL on days 1, 3 and 6 after DSS treatment. The control group was given the same amount of saline physiological solution via intraperitoneal injection. The treatment period lasted for 7 days (Figure 4A).

### 5.8 DAI score

The general physiological conditions of the mice, including eating, drinking, motor activity, body weight, hair condition and stool characteristics, were observed before and after treatment. Occult blood in the stool was detected using faecal test paper. The DAI was evaluated using the criteria outlined in Table S1.

### 5.9 Direct observation of colon tissue

On day 7, all mice were euthanized, and the abdominal wall was incised along the midline to expose the underlying tissues and the colon. The morphology and length of the entire intestine were compared. Colon segments were excised, and the adipose mesenchymal tissue was removed. The colon tissue was homogenized in 10 volumes of ice‐cold saline buffer. Myeloperoxidase (MPO) activity was measured using a specific kit according to the manufacturer's protocol (A044‐1‐1, Nanjing Jiancheng Bioengineering Institute). Additionally, the levels of TNF‐α, IFN‐γ, IL‐23 and IL‐17 cytokines were measured by ELISA using the following kits: TNF‐α (EM008, Excell Bio), IL‐23 (CSB‐E08463, Cusabio), IFN‐γ (EM007, Excell Bio) and IL‐17 (EM015, Excell Bio).

### 5.10 Total RNA extraction and quantitative real‐time PCR

Utilizing TRIzol reagent (Invitrogen), total RNA was extracted, and reverse transcriptase kit (Invitrogen) was used to synthesize cDNA in accordance with the manufacturer's instructions. Using a Roche LightCycler 480 II device, quantitative real‐time PCR amplification was performed using SYBR green premix ExTaq. Table S2 contains the primer sequences unique to the target genes.

### 5.11 IHC tests

Paraffin sections (5 µm thickness) were heated at 58°C for 1−2 h, washed in water, and then covered using a solution for antigen repair pH 6.0, .01 M citric acid before incubating in a pressure cooker at 90°C for 2 min. In order to inhibit endogenous peroxidase activity, the slices were incubated with 3% H2O2 for 30 min. Slides were then washed with PBS‐T and incubated with goat serum for 2 h. Subsequently, the sections were treated with primary antibodies for an entire night at 4°C: anti‐CD4 antibody (1:2000), anti‐F4/80 antibodies (1:500) and anti‐MPO antibody (1:1000), all diluted with PBS (pH 7.5). After incubation, the sections were equilibrated to room temperature for 15 min and then incubated with polyβ‐hydroxybutyrate for 15 min. Slides were dried and incubated with goat anti‐rabbit IgG (H+L) secondary antibody (31210, Invitrogen) at a dilution of 1:10 000 for 30 min at room temperature. Colour development was induced by adding 3,3′‐diaminobenzidine (DAB, Beyotime Biotech), and the reaction was observed under a microscope. The reaction was stopped by immersing the samples in water. A haematoxylin dye solution was applied for 2 min. Subsequently, the samples were treated with hydrochloric acid alcohol to enhance contrast, thoroughly rinsed with double‐distilled water, dehydrated in absolute ethanol, sealed, and observed and photographed using a charge‐coupled device (CCD) microscope. Histological scoring of inflammation was independently performed by two senior pathologists.

Twenty‐four SPF female C57BL/6J mice were randomly divided into four groups: a control group (untreated), a DSS model group, a DSS+BsNb‐Fc group and a DSS+anti‐mouse TNF‐α mAb group. The anti‐mouse TNF‐α mAb was purchased from Bioxcell. The modelling with DSS and the antibody treatment methods were carried out as described above.

### 5.12 In vitro anti‐inflammatory and apoptotic actions of BsNb‐Fc

Raw264.7 and THP‐1 cells were cultured in culture media (CM‐0233, Procell). RAW264.7 cells were plated in 96‐well plates at a density of 5×10^3^ cells/mL, and they were incubated for an entire night at 37°C with 5% CO2. The cells were then stimulated with LPS (.1 µg/mL) for 6 h. This procedure was repeated using various doses of BsNb‐Fc (.1, .5, 1, 5 and 10 µg/mL). After an additional 18‐h incubation, CCK‐8 reagent (C0038, Beyotime) was added at a volume of 10 µL per well and incubated for four more hours. Every sample's absorbance was calculated at OD450 using GraphPad Prism software to calculate cell survival rates. ELISA assays were performed to measure changes in TNF‐α (430907, Biolegend), IL‐6 (431307, Biolegend) and IL‐1β (E‐EL‐M0037c, Elabscience) levels among different experimental groups, including control (non‐induced), LPS alone, LPS+BsNb‐Fc, LPS+IFX&UST, LPS+IFX and LPS+UST.

Phorbol myristate acetate (PMA) was used to drive THP‐1 cells to develop into macrophages, and these cells were subsequently polarized into M1 macrophages by LPS and IFN‐γ, resulting in the discharge of cytokines like IL‐6 and TNF‐α, thus establishing a typical inflammation model.^68^ Ninety‐six‐well plates were seeded with 8×10^4^ THP‐1 cells per millilitre and incubated for a full night. They were then stimulated with 50 ng/mL PMA for 18 h. After incubation, the cells were washed twice and fresh media (without PMA) were added for an additional 24 h. The media were then replaced, and the cells were treated with LPS (1 µg/mL) and different drug treatment groups (same as RAW264.7) for 18 h. Following incubation, the same assay was performed.

In order to look into how BsNb‐Fc regulates the interaction between macrophages and CD4^+^ T cells, RAW264.7 was co‐incubated with mouse CD4^+^ T cells. The activation of CD4^+^ T cells and changes in secreted cytokines were then measured. Using a magnetic bead sorting kit (Miltenyi Biotec), naïve CD4^+^T cells were extracted from a single‐cell suspension of C57BL/6J mouse spleen and adjusted to a concentration of 2×10^6^ cells/mL in TexMACS medium supplemented with 10% FBS (Miltenyi Biotec). A 24‐well cell culture plate was used, and CD4^+^ T cells (2×10^6^ cells/mL, 1 mL) were added to each well, with three replicate wells per group. Then, 20 µL of CD3/CD28‐loaded MACS beads (Miltenyi) were added per 2×10^6^ cells and incubated at 37°C with 5% CO₂ for 4 days. On day 1, Th1 cells were induced (10 ng/mL IL‐12, 10 ng/mL IL‐2, 10 µg/mL IL‐4 antibody, Miltenyi) and Th17 cells were induced (20 ng/mL IL‐6, 10 ng/mL IL‐23, 10 ng/mL IL‐1β, 2 ng/mL TGF‐β, 10 µg/mL IL‐4 antibody, 10 µg/mL IL‐2 antibody, 10 µg/mL IFN‐γ antibody, Miltenyi) in separate wells. On the second day, the culture was gently mixed to dislodge the aggregated cells. On day 3, was split at a ratio of 1:2 with fresh medium added, and Th1 cells were supplemented with IL‐2 (10 ng/mL). On day 5, differentiated T cells were washed and co‐cultured with LPS‐stimulated RAW264.7 cells in a 24‐well plate, with different drug treatment groups: 1 µg/mL BsNb‐Fc or 1 µg/mL IFX&UST. Cells were re‐stimulated using the same activation method as during induction, and supernatants were collected after 24 h for ELISA analysis to detect Th1‐ and Th17‐related cytokines, including IFN‐γ and IL‐2 (Biolegend) for Th1 cells, and IL‐17A and IL‐22 (Elabscience) for Th17 cells.

### 5.13 Co‐culturing THP‐1 cell culture supernatant with human CD4^+^ T cells

Naïve CD4^+^ T cells were extracted from the peripheral blood of healthy individuals using anti‐human naïve CD4 magnetic beads from BD Biosciences. Cells were stimulated with plate‐coated anti‐CD3 mAb (5 µg/mL; eBioscienc) and anti‐CD28 mAb (2 mg/mL; eBioscience) in full RPMI 1640 medium at a concentration of 5×10^5^ cells per well.

Upon the recovery of THP‐1 cells and entering the logarithmic growth phase, the cells were placed onto 24‐well plates. Concurrently, 50 ng/mL of PMA was introduced, and the cells were incubated for another 24 h. After discarding the supernatant, either 1 µg/mL LPS or 1 µg/mL LPS+10 µg/mL BsNb‐Fc was added. The cultures were maintained for 24 h, and then the liquid above the sediment was extracted. The LPS group was not given more LPS to prevent its stimulatory effect on CD4^+^ T cells, while the BsNb‐Fc group received BsNb‐Fc for an additional 24 h. THP‐1 cell culture supernatant was harvested and introduced to CD4^+^ T cells.

### 5.14 Flow cytometry

CD4+ T cells were obtained after culture. The cells were cultured with PMA (50 ng/mL) and ionomycin (1 µg/mL; Sigma‐Aldrich) for 5 h, with brefeldin A (3 µg/mL; eBioscience) added for the last 3 h in 10% FBS‐RPMI media at 37°C. The cells were fixed and permeabilized for 30 min at 4°C. Cells were then stained with fluorochrome‐conjugated antibodies against IFN‐γ, IL‐17A and TNF‐α from BD Biosciences. The data were obtained using FACSCanto II (BD Biosciences) and processed with Flowjo software.

### 5.15 Establishment of TNBS‐induced colitis in mice and BsNb‐Fc treatment

Colitis was induced in SPF C57BL/6J mice by rectal administration of 2.5% TNBS (Sigma) in 50% ethanol following cutaneous pre‐sensitization. One week before the challenge, a 2×2 cm area of abdominal skin was shaved, and 100 µL of 1% TNBS was applied for sensitization.^41^ On day 8, 100 µL of 2.5% TNBS was injected approximately 3.5−4 cm into the mouse's anus. Mice were then held vertically for 1 min to ensure the distribution of TNBS throughout the colon and cecum. Different drugs were administered 24 h after TNBS administration for 3 consecutive days until day 7 (Figure 6A).^39^ Experimental animals were divided into seven groups (six mice per group): control (50% ethanol), TNBS+PBS, TNBS+BsNb‐Fc (5 mg/kg), TNBS+IFX (2.5 mg/kg) & UST (.25 mg/kg), TNBS+IFX (5 mg/kg), TNBS+UST (.5 mg/kg) and TNBS+ISO (human IgG1, kappa isotype control; Sino Biological). MPO activity was measured using an MPO activity assay kit (E‐BC‐F013, Elabscience).

### 5.16 Statistical analyses

The mean ± standard error of the mean was used to express the findings.^69^ Software called GraphPad Prism 8.4.3 was used to analyse the data, and was calculated using a ordinary one‐way ANOVA (and Nonparametric or mix).^9^ Statistical significance was defined as a *p*‐value < .05.

## AUTHOR CONTRIBUTIONS

**Study design**: Zhanju Liu; Xiaocang Cao; He Huang; Huahong Wang and Xiaolan Zhang. **Literature search**: Jiewen Wang; Guangbo Kang; Yuli Wang and Miao Zhang. **Data collection**: Jiewen Wang; Huiying Lu; Mengxue Gao and Xiaoli Wang. **Data analysis**: Jiewen Wang; Huiying Lu; Guangbo Kang; Haibin Yuan and Zelin Feng. **Data interpretation**: Jiewen Wang; Huiying Lu; Guangbo Kang; Ario de Marco; Xiaocang Cao and He Huang. **Figures**: Jiewen Wang and Zelin Feng. **Revise**: Jiewen Wang; Guangbo Kang; Ario de Marco; Zhanju Liu; Xiaocang Cao and He Huang.

## CONFLICT OF INTEREST STATEMENT

The authors declare that they have no conflict of interest.

## ETHICS STATEMENT

In this study, we strictly adhered to ethical requirements for experimental animals and established clear humane endpoints. During the course of the experiment, some mice in the untreated group experienced severe health issues, including weight loss, bloody stools and motor impairment. To protect the welfare of the animals, we immediately terminated these experiments and performed euthanasia to alleviate their suffering. This practice reflects our respect for the lives of the experimental animals while ensuring the scientific validity of the experimental data.

## Supporting information

Supporting Information

## Data Availability

Data are available upon reasonable request by sending a message to the corresponding author.
