# Supplementary material for: Novel bispecific nanobody mitigates experimental intestinal inflammation in mice by targeting TNF‐α and IL‐23p19 bioactivities
Source: Clin Transl Med. 2024 Mar 27;14(3):e1636. doi: 10.1002/ctm2.1636 (PMC10966562; doi:10.1002/ctm2.1636)
Supplement: Supplementary file 1 — Supporting Information [file CTM2-14-e1636-s001.doc]

Table S1： DAI scoring criteria

| Lost weight（%） | stool property | Fecal occult blood  /gross bloody stool | score |
| --- | --- | --- | --- |
| 0 | Normal | Normal | 0 |
| 1-5 | Loose | OB+ | 1 |
| 5-10 |  |  | 2 |
| 10-15 | Pasty | Macroscopic | 3 |
| >15 |  |  | 4 |

Stool occult blood (OB) test was performed by smearing mouse feces on OB test paper and considering the time of color change and its intensity.

Normal stool: formed stool; Loose stool: mushy, semi-formed stool that does not adhere to the anus; Pasty stool: watery stool that adheres to the anus.

Table S2：Gene-specific primer sequences for qPCR

| Primer name | sequences |
| --- | --- |
| GAPDH-F | TGTGTCCGTCGTGGATCTGA |
| GAPDH-R | TTGCTGTTGAAGTCGCAGGAG |
| IL-1β-F | ACATGCACCAGCGGGACATA |
| IL-1β-R | CTTTGAAGATGTCAGAGTCAAGCAG |
| IL-2-F | CTTGCCCAAGCAGGCCACAG |
| IL-2-R | GAGCCTTATGTGTTGTAAGC |
| IL-4-F | GAATGTACCAGGAGCCATATC |
| IL-4-R | CTCAGTACTACGAGTAATCCA |
| IL-6-F | TGCTGGTGACAACCACGGCC |
| IL-6-R | GTACTCCAGAAGACCAGAGG |
| IL-10-F | CCAAGCCTTATCGGAAATGA |
| IL-10-R | TTCACAGGGGAGAAATCG |
| IL-17-F | ACGCGCAAACATGAGTCCAG |
| IL-17-R | AGGCTCAGCAGCAGCAACAG |
| IFN-γ-F | CGGCACAGTCATTTGAAAGCCTA |
| IFN-γ-R | GTTGCTGATGGCCTGATTGTC |
| TGF-β-F | CCCTGGATACCAACTATTGC |
| TGF-β-R | GCAGAAGTTGGCATGGTAGC |
| NF-κB-F | TCAATGGCTACACAGGACCA |
| NF-κB-R | ATCTTGAGCTCGGCAG |
| STAT3-F | CATCGCTTGCGGCAGTC |
| STAT3-R | CGTACTGCGTGCGATGA |
| IL23 F | CAGCAGCTCTCTCGGAATCTC |
| IL23 R | TGGATACGGGGCACATTATTTTT |
| TNF-α F | CCTGTAGCCCACGTCGTAG |
| TNF-α R | GGGAGTAGACAAGGTACAACCC |
| IL-22 F | ATGAGTTTTTCCCTTATGGGGAC |
| IL-22 R | GCTGGAAGTTGGACACCTCAA |

Table S3：Characteristic features of the different constructs binding to TNF-α and IL-23.

| Nanobody constructs | Yield (mg/L) | Tm (℃) | Affinity to TNF-α  (nM) | | Affinity to IL-23  (nM) |
| --- | --- | --- | --- | --- | --- |
| VHH#1 | 10.0 | 69.37 | | 5.31 | / |
| VHH#2 | 10.6 | 53.80 | | 4.42 | / |
| VHH#37 | 17.3 | 55.76 | | / | 7.25 |
| VHH#22 | 15.0 | 50.06 | | / | 6.17 |
| VHH#2-9GS-VHH#22 | inclusion bodies | nd | | nd | nd |
| VHH#2-15GS-VHH-37 | inclusion bodies | nd | | nd | nd |
| VHH#2-9GS-VHH#37 | inclusion bodies | nd | | nd | nd |
| VHH#37-15GS-VHH#2 | 13.5 | 55.13 | | 4.31 | 9.92 |
| VHH#37-9GS-VHH#1 | 7.4 | 46.12 | | 10.35 | 22.14 |
| VHH#37-15GS-VHH#1 | 6.1 | 44.05 | | 8.56 | 28.66 |
| VHH#22-9GS-VHH#1 | 12.4 | 42.87 | | 10.1 | 15.29 |
| VHH#22-15GS-VHH#1 | 9.7 | 44.91 | | 9.15 | 19.37 |
| VHH#2-15GS-VHH#22 | 8.6 | 47.96 | | 10.61 | 15.02 |
| VHH#22-9GS-VHH#2 | 7.8 | 44.40 | | 10.84 | 16.84 |
| VHH#22-15GS-VHH#2 | 6.9 | 48.47 | | 6.13 | 24.42 |
| VHH#37-9GS-VHH#2 | 6.3 | 43.01 | | 9.72 | 21.9 |
| VHH#1-9GS-VHH#37 | 8.6 | 44.56 | | 11.31 | 11.68 |
| VHH#37-15GS-VHH#2-Fc | 50 | 60.55 | | 6.23 | 0.59 |

Tm and affinity values were calculated when soluble constructs were available. nd, not determined.


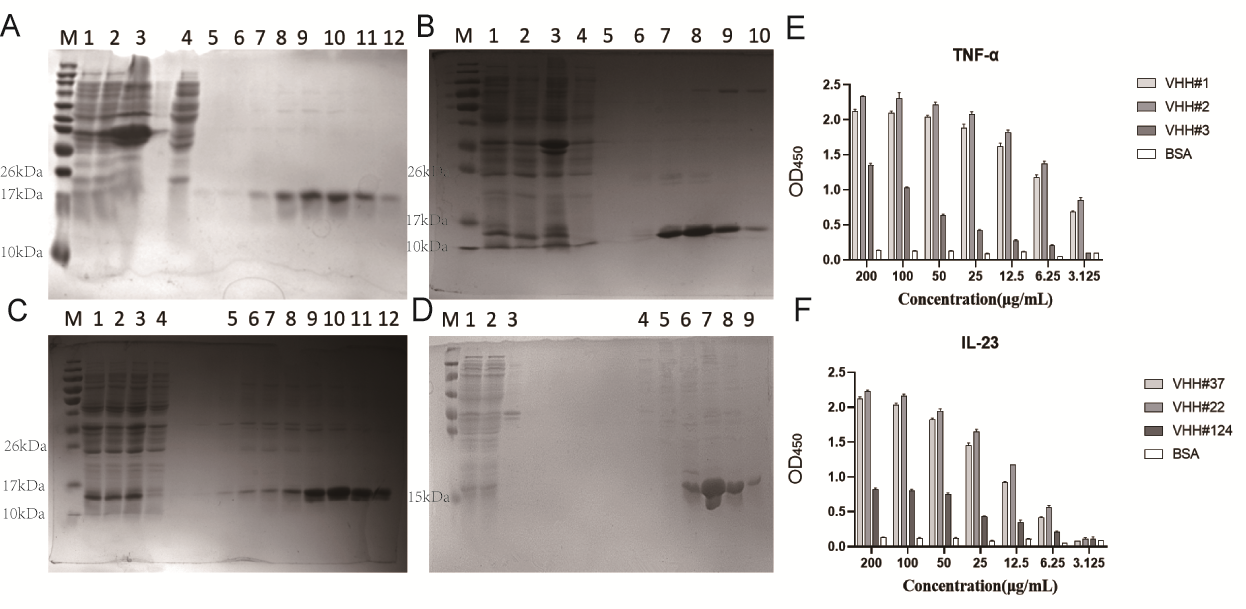


**Figure S1**. Purification and functional characterization of TNF- and IL-23-specific VHHs. (A)VHH#1 (anti-TNF-α) (B)VHH#2 (anti-TNF-α) (C and D)VHH#22 (anti-IL-12&IL-23 p40) and VHH#37 (anti-IL-23 p19) were expressed in E. coli transB. Lanes 1-4 correspond to bacterial lysate, supernatant, pellet, and flow-through, respectively. Lanes 5-12 indicate elution fractions. E and F demonstrate VHH binding activity to tested antigens assessed by ELISA.


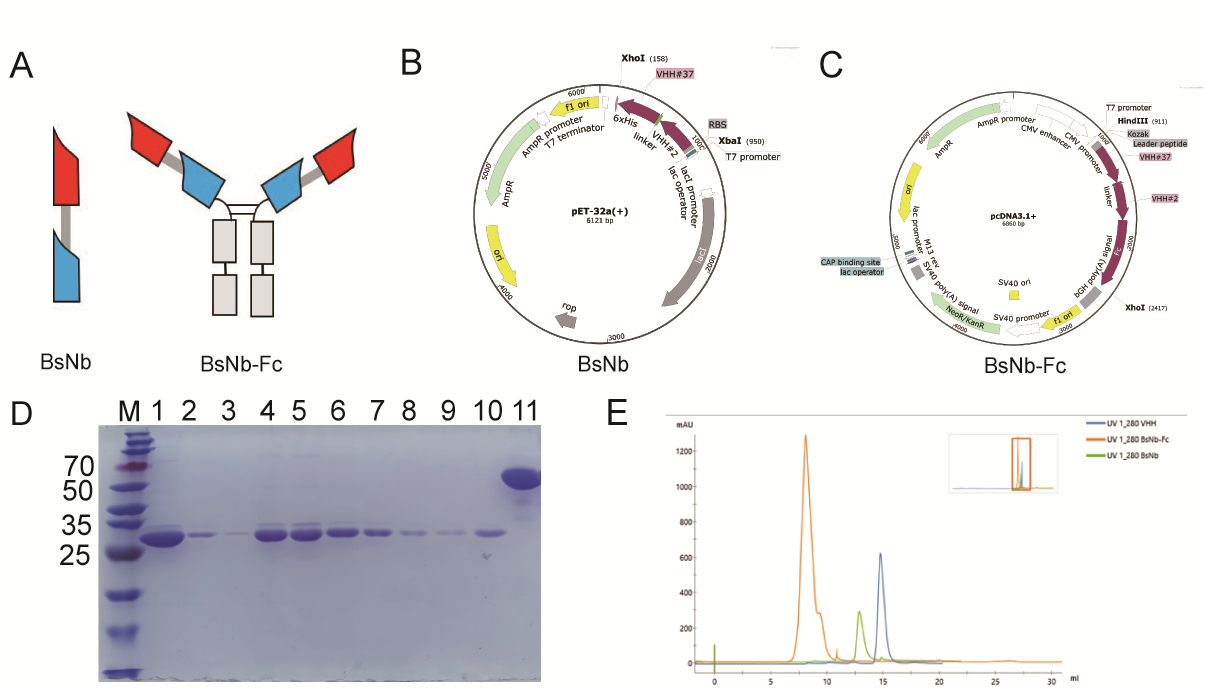


**Figure S2**. Expression and purification of bispecific nanobodies. (A) Schematic representation of representative bispecific nanobodies, BsNb and BsNb-Fc. The red domain represents an anti-IL-23 VHH, while the blue domain represents an anti-TNF-α VHH. (B and C) Structural diagrams of BsNb and BsNb-Fc expression plasmids. (D) SDS-PAGE (12%) analysis of BsNb purified from E. coli (32 kDa, lanes 1-10) and BsNb-Fc purified from HEK293 cells (55 kDa, lane 11), respectively. M: protein markers. (E) Size-Exclusion Chromatography profiles of the purified constructs VHH#2, VHH#37-15GS-VHH#2, and VHH#37-15GS-VHH#2-Fc, respectively.


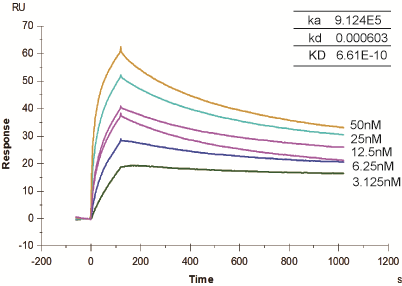


**Figure S3**. Binding affinity analyses of IFX to TNF-α.


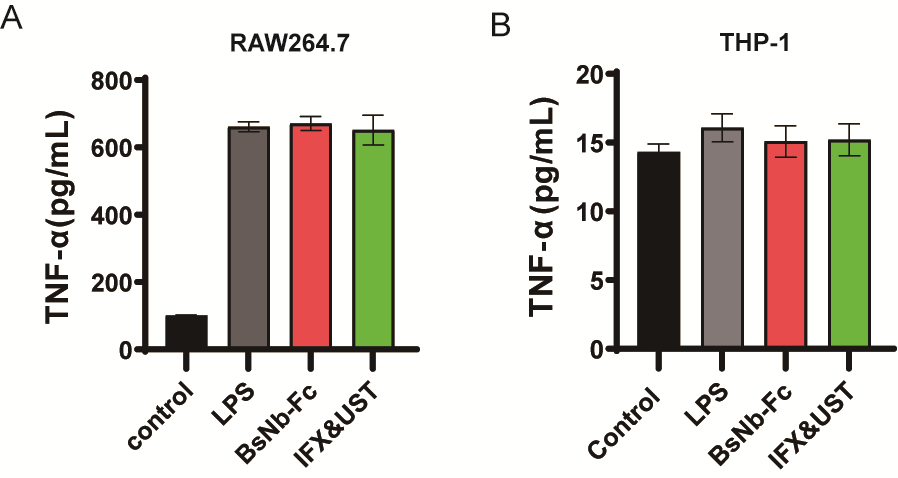


**Figure S4**. Stimulation of RAW264.7 and THP-1 macrophage cells with varying concentrations of lipopolysaccharide. (A) Following stimulation of RAW264.7 cells with 1μg/mL LPS and subsequent addition of 10μg/mL antibodies, TNF-α levels in the medium were quantified using ELISA. (B) Following stimulation of THP-1 cells with 0.1μg/mL LPS and subsequent addition of 10μg/mL antibodies, TNF-α levels in the medium were quantified using ELISA.


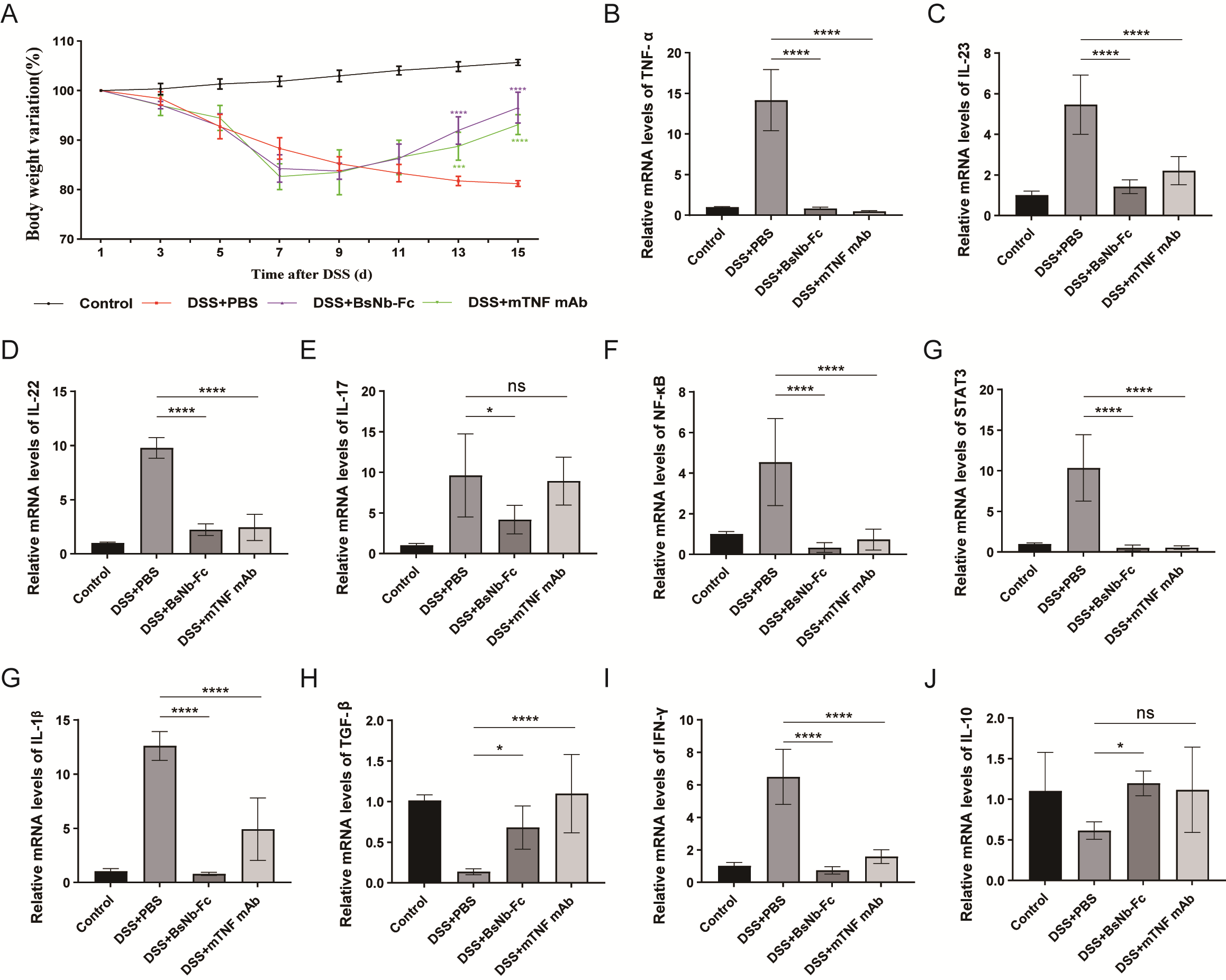


**Figure S5. BsNb-Fc and anti-mouse TNF-α mAb treatments modulate cytokine expression induced by DSS in the colon tissue.** A. Body weight variation curves depicting the changes in in body weight of mice treated with antibodies compared to the DSS+PBS. B-J The mRNA expression levels of various pro-inflammatory cytokines, including TNF-α, IL-23, IL-22, IL-17, and IL-1β, as well as anti-inflammatory cytokines IL-10 and TGF-β, were assessed. Additionally, the mRNA expression levels of NF-κB and STAT3 were measured. Data are presented as the means±SEM and were analyzed using ordinary one-way ANOVA with multiple comparisons. Statistical significance is denoted as *P < 0.05, **P < 0.01, ***P < 0.001, ****P < 0.0001, compared with the respective DSS+PBS group.


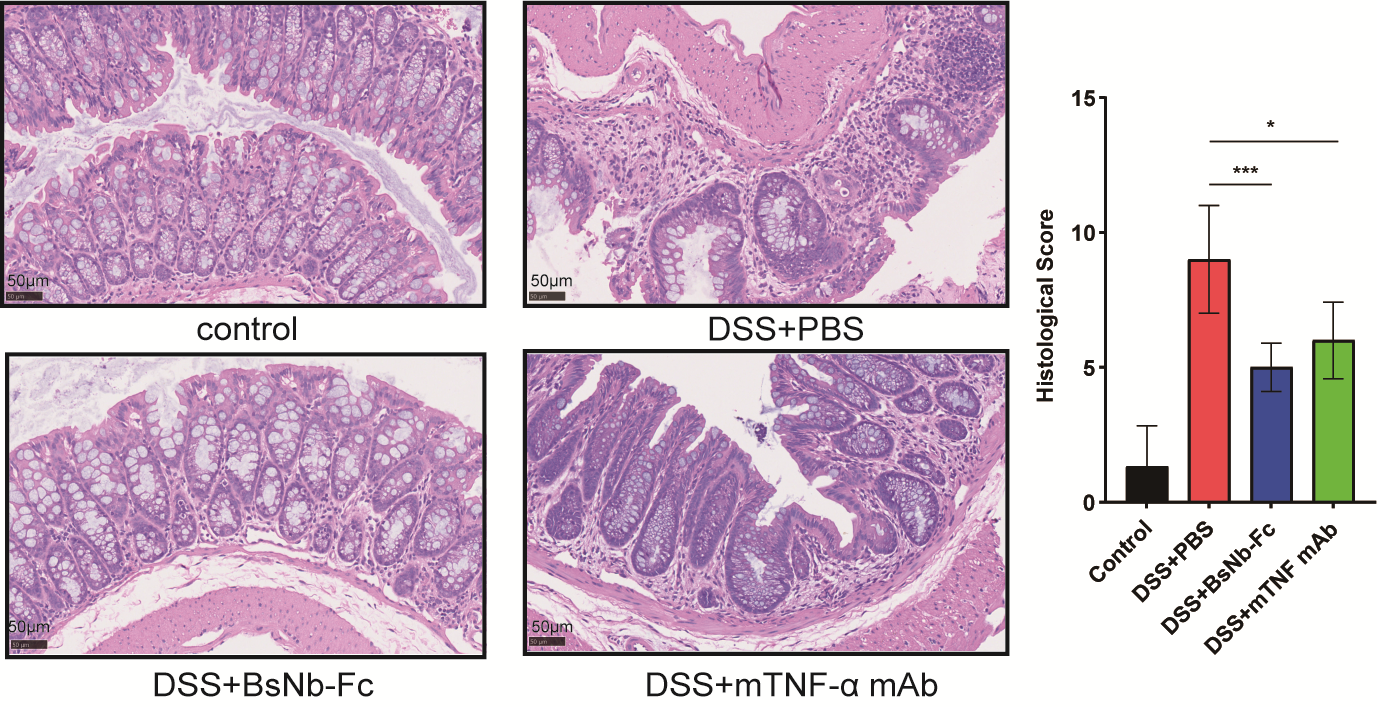


**Figure S6. Representative histological images of colon tissues stained with H&E, with corresponding calculation of pathological score to assess colonic tissue damage.** Scale bars, 50 µm.
